# Association between Combined Structure Function Index and Glaucoma Severity

**DOI:** 10.1155/2019/9414675

**Published:** 2019-03-25

**Authors:** Shumpei Ogawa, Yoshimasa Tanabe, Yoshinori Itoh, Takahiko Noro, Hisato Gunji, Tadashi Nakano

**Affiliations:** ^1^Department of Ophthalmology, The Jikei University School of Medicine, 3-25-8 Nishishimbashi, Minato, Tokyo 105-8461, Japan; ^2^Department of Ophthalmology, Atsugi City Hospital, 1-16-36 Mizuhiki, Atsugi, Kanagawa 243-8588, Japan; ^3^Department of Ophthalmology, Stanford University, 2452 Watson Ct., Palo Alto, CA 94303, USA

## Abstract

The aim of this study was to investigate the relationship between combined structure function index (CSFI) and standard automated perimetry (SAP) parameters such as mean deviation (MD) and visual field index (VFI) in open-angle glaucoma (OAG). We retrospectively reviewed medical records from September 2009 to July 2015, which included 195 eyes of 195 patients with OAG or normal-tension glaucoma who underwent SAP and optical coherence tomography on the same day (male: female, 128 : 67; mean age, 61.4 ± 11.3 years; mean spherical equivalent, −2.39 ± 2.3 D). We divided participants into three stages based on MD value: early, MD > −6 dB; middle, −6 dB ≥ MD ≥ −12 dB; and advanced, MD < 12 dB. We then evaluated correlations between CSFI and SAP parameters in each stage using Pearson's correlation coefficient. Mean CSFI (%), mean MD (dB), and VFI (%) in each stage were early (22.4, −2.13, and 94.0); middle (47.9, −8.78, and 75.4); and advanced (68.3, −17.32, and 49.0), respectively. Correlations between CSFI and whole, early, middle, and advanced MD were −0.88 (*p* < 0.001), −0.68, −0.24, and −0.76, respectively. Correlations between CSFI and whole, early, middle, and advanced VFI were −0.86 (*p* < 0.001), −0.59, −0.20, and −0.83, respectively. Consistency between CSFI and SAP indices in middle-stage glaucoma was low.

## 1. Introduction

Glaucoma is a progressive optic neuropathy with characteristic structural changes in the optic nerve head reflected in the visual field and represents the second most common cause of irreversible blindness worldwide [1]. In clinical settings, the current gold standard for glaucoma is diagnosis by combining optic disc assessment for structural changes and achromatic white-on-white perimetry to monitor visual field defects [2]. Optical coherence tomography (OCT) has recently played important roles in glaucoma diagnosis, monitoring of progression, and quantification of structural damage [3].

Harwerth et al. [4] showed that glaucomatous neural damage was predictable from measurements of visual sensitivity using clinical perimetry in a rhesus monkey glaucoma model. Medeiros et al. [5] modified the Harwerth model and added the retinal nerve fiber layer thickness as measured by OCT to suit human glaucoma patients, creating what they called the combined structure function index (CSFI). The utility of CSFI in achieving early diagnosis [6, 7] and predicting disease progression [8] has recently been reported.

Standard automated perimetry (SAP) is currently used as a standard tool for diagnosing and staging disease, but several studies have shown that considerable numbers of retinal ganglion cells (RGCs) are already lost prior to visual function loss detectable on SAP [4, 9–15]. Compared to OCT, SAP is suitable for monitoring glaucoma progression from the middle to advanced stage. SAP reliability in the very advanced stage (mean deviation, MD < −20 dB) may be low because of a reduction in the asymptotic maximum response probability [16].

The present study evaluated the relationship between CSFI and the SAP parameters of MD and visual field index (VFI). No detailed reports have described CSFI in each glaucoma stage or correspondence with the glaucoma field of vision index. We found a low correlation coefficient between CSFI and SAP index in middle-stage glaucoma. Our results will aid in the understanding of CSFI variability and inform researchers and clinicians about the ability to monitor glaucoma progression.

## 2. Materials and Methods

We retrospectively reviewed the medical records of patients with primary open-angle glaucoma (OAG) or normal-tension glaucoma who underwent SAP and OCT on the same day in the Glaucoma Unit, Department of Ophthalmology, Jikei University School of Medicine, Tokyo, Japan, from September 2009 to July 2015. All participants underwent a comprehensive ophthalmic examination including measurements of best-corrected visual acuity, refraction, intraocular pressure using a Goldmann applanation tonometer, gonioscopy, and OCT and SAP measurement (Tables 1 and 2). This study was conducted according to the principles of the Declaration of Helsinki, and the Jikei University Hospital Ethics Committee approved the study protocol (no. 25–172).

### 2.1. Inclusion Criteria

Inclusion criteria were as follows:

1. Patients with primary OAG (POAG) and normal-tension glaucoma (NTG).
2. Standard automated white-on-white perimetry was performed using the Swedish interactive threshold algorithm standard 30–2 program in the Humphrey Field Analyzer II-i (Carl Zeiss Meditec, Dublin, CA). A reliable VF test was considered to have fixation losses <20%, false-positive errors <15%, and false-negative errors <33%. A VF defect showed ≥3 significant (*p* < 0.05) nonedge contiguous points with at least 1 point at the *p* < 0.01 level on the same side as the horizontal meridian in the pattern deviation plot [17].
3. Cirrus HD-OCT (software version 9.5; Carl Zeiss Meditec) measured retinal nerve fiber layer (RNFL) thicknesses from the optic disc cube. The protocol is based on a tridimensional scan of a 6 × 6 mm^2^ area centered on the optic disc where information from a 1024 (depth) × 200 × 200 point parallelepiped is collected. Then, a 3.46 mm circular scan is placed around the optic disc, and the information about peripapillary RNFL thickness is obtained. All OCT scans showed signal strength ≥6. OCT showing motion artifacts, poor centration, or missing data were discarded, and rescanning was performed in the same visit.
4. No other eye disease (e.g., epiretinal membrane and macular edema), excluding cataract grade less than Emery-Little 2 [18] or intraocular lens.
5. No general disease (e.g., diabetes mellitus and encephalopathy affecting visual field).
6. Spherical equivalent power > −6.

Finally, 195 eyes in 195 subjects (high-tension glaucoma (HTG) : NTG, 99 : 96 eyes) were studied. We then classified patients into three groups according to glaucoma stage: early, MD > −6 dB (104 eyes); middle, −6 dB ≥ MD ≥ −12 dB (42 eyes); and advanced stage, MD < 12 dB (49 eyes).

### 2.2. Estimation of CSFI

**Figure 1:**
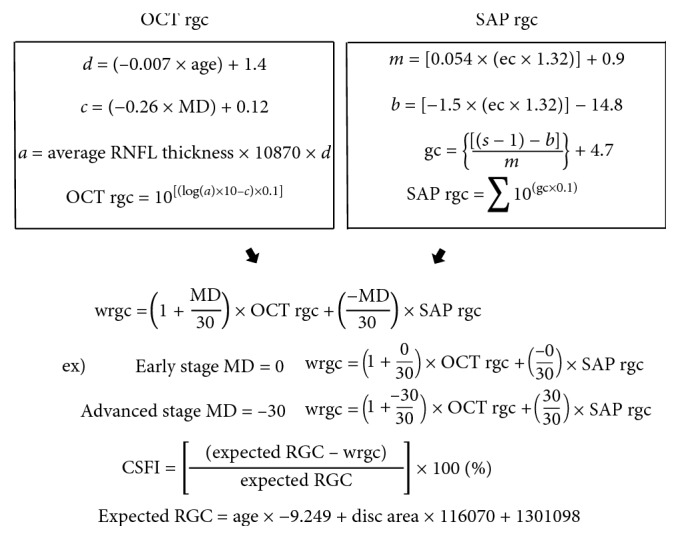
Formula for calculating CSFI and SAP rgc is obtained from the eccentricity (degrees) and OCT rgc from age (years) and MD. Values of SAP rgc and OCT rgc thus obtained are balanced by MD to calculate wrgc. OCT rgc accounts for a greater proportion in the early stage and SAP rgc for a greater proportion in the advanced stage. Age-corrected normal RGC (expected RGC), the denominator of CSFI, is calculated from the disc area (mm^2^) on OCT and age, so that the rate of loss of wrgc is expressed as the CSFI (%). SAP, standard automated perimetry; OCT, optical coherence tomography; RGC or rgc, retinal ganglion cell; CSFI, combined structure function index; MD, mean deviation; RNFL, retinal nerve fiber layer.

CSFI was calculated in this study using an identical formula to that developed for Medeiros et al. [5]. We briefly describe the major details of CSFI in Figure 1.

### 2.3. Statistical Analysis

Pearson's correlation coefficient was calculated to evaluate the relationship between CSFI and SAP parameters (MD and VFI) in whole and each glaucoma stage. Analysis of covariance (ANCOVA) was used to see whether glaucoma disease type affects the relationship between CSFI and SAP parameters. Analysis of variance (ANOVA) was conducted for numerical scale more than 3 groups comparison and Wilcoxon rank-sum test for numerical scale 2 group comparison. Fisher's exact test was calculated for ratio scale group comparison. Bland and Altman analysis [19] was used to estimate agreement between SAP rgc and OCT rgc. The method only defines the intervals of agreements; it does not provide whether those limits are acceptable or not [20]. Descriptive statistics are provided as mean ± standard deviation. The statistical analysis of all the data were performed using Matlab software (ver. 2015b; MathWorks, Natick, MA) and R software (ver. 3.3.1, http://www.R-project.org) [21]. Values of *p* < 0.05 were considered statistically significant.

## 3. Results

Finally, 195 eyes of 195 subjects (POAG : NTG, 99 : 96 eyes) were studied (see Section 2.1). Demographic and clinical characteristics of the study are illustrated in Table 1.

Parameters are given as mean ± standard deviation. Group difference was tested using ANOVA for a numerical scale^*∗*^ and Fisher's exact test for a ratio scale^*∗∗*^. Statistical significance was set at *p* < 0.05^#^. CSFI, combined structure function index; MD, mean deviation; VFI, visual field index; SE, spherical equivalent.

### 3.1. Correlation between CSFI and SAP Parameters

**Figure 2:**
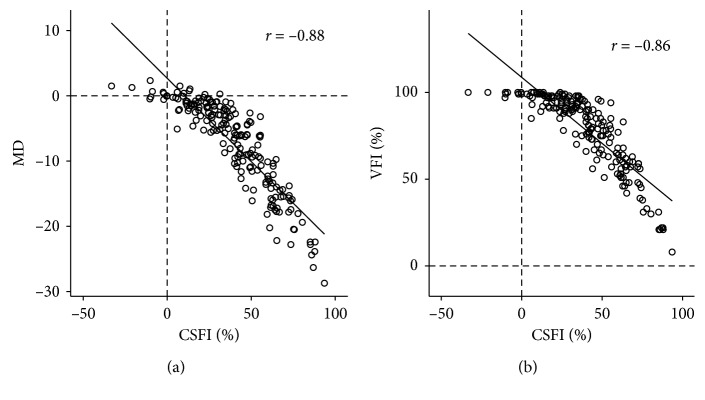
Relationship between CSFI and SAP parameters with all subjects. (a) Scatter plot showing correlation between CSFI and MD (*r* = −0.88, *p* < 0.001). (b) CSFI and VFI (*r* = −0.86, *p* < 0.001). Dotted line indicates MD = 0 dB and VFI = 0%.

All patients were distributed in CSFI 39.4 ± 23.7%, MD −7.38 ± 6.84 dB, and VFI 78.7 ± 21.0%, respectively (Table 1). We found the significant correlation between CSFI and SAP parameters such as MD and VFI, between CSFI and MD (*r* = −0.88, *p* < 0.001) and between CSFI and VFI (*r* = −0.86, *p* < 0.001) (Figure 2). The VFI cannot increase above 100%. This may cause a ceiling effect on the correlation between CSFI and VFI. To see this effect, we exclude subjects who have VFI = 100%; after exclusion, the correlation coefficient slightly increased from −0.86 to −0.84 (*p* < 0.001) (not shown in Figure 2).

### 3.2. Correlation between CSFI and SAP Parameters in Each Stage

Mean CSFI (%) in each stage was as follows: early, 22.4 ± 16.2; middle, 47.9 ± 7.7; and advanced, 68.3 ± 10.9, respectively. Mean MD (dB) and VFI (%) in each stage were −2.13 ± 1.99 and 94.0 ± 5.5, −8.78 ± 1.78 and 75.4 ± 8.7, and −17.32 ± 3.90 and 49.0 ± 15.2, respectively. SE, MD, VFI, and CSFI were significantly different (Table 1).

**Figure 3:**
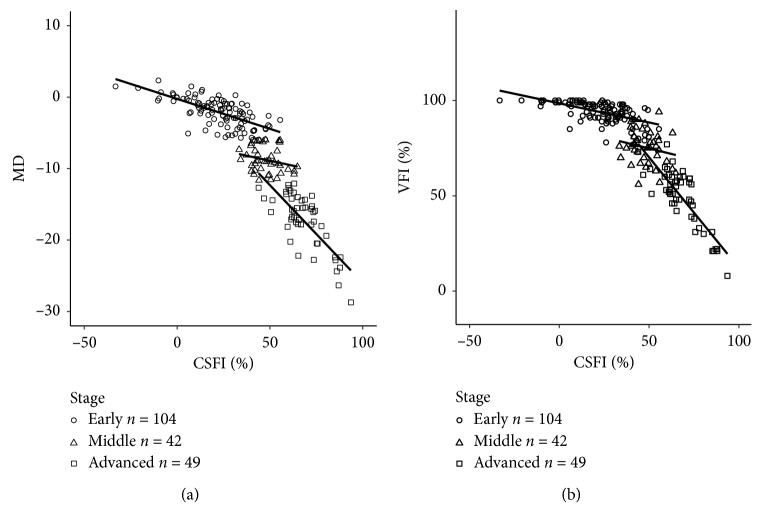
Relationship between CSFI and MD and VFI (early *n* = 104, middle *n* = 42, advanced *n* = 49) in a scatter plot. (a) Correlation between CSFI and MD: early *r* = −0.68 (*p* < 0.001), middle *r* = −0.24 (*p* < 0.1239), and advanced *r* = −0.79 (*p* < 0.001). (b) Correlation between CSFI and VFI: early *r* = −0.59 (*p* < 0.001), middle *r* = −0.20 (*p* < 0.1943), and advanced *r* = −0.83 (*p* < 0.001).

Figure 3 shows the relationship of CSFI with MD and VFI in a scatter plot at each stage (early *n* = 104, middle *n* = 42, and advanced *n* = 49).

In the early stage, CSFI vs. MD: *r* = −0.68, *p* < 0.001 and CSFI vs. VFI: *r* = −0.59, *p* < 0.001; in the middle stage, CSFI vs. MD: *r* = −0.24, *p*=0.1239 and CSFI vs. VFI: *r* = −0.20, *p*=0.1943; in the advanced stage, CSFI vs. MD: *r* = −0.76, *p* < 0.001 and CSFI vs. VFI: *r* = −0.83, *p* < 0.001. We exclude subjects who have VFI = 100% to see a ceiling effect on the correlation between CSFI and VFI in the early stage; the correlation coefficient slightly increased from −0.59 to −0.52 (*p* < 0.001) (not shown in Figure 3).

### 3.3. Investigation between CSFI and SAP Parameters for Disease Type

No significant difference between POAG and NTG at all parameters in mean age, SE, sex, MD, VFI, and CSFI (Table 2).

The parameters are mean and standard deviation. Significance was tested using the Wilcoxon rank-sum test for numerical scale^*∗*^ and Fisher's exact test for ratio scale^*∗∗*^. Statistical significance was set at *p* < 0.05. CSFI, combined structure function index; MD, mean deviation; VFI, visual field index; SE, spherical equivalent.

**Figure 4:**
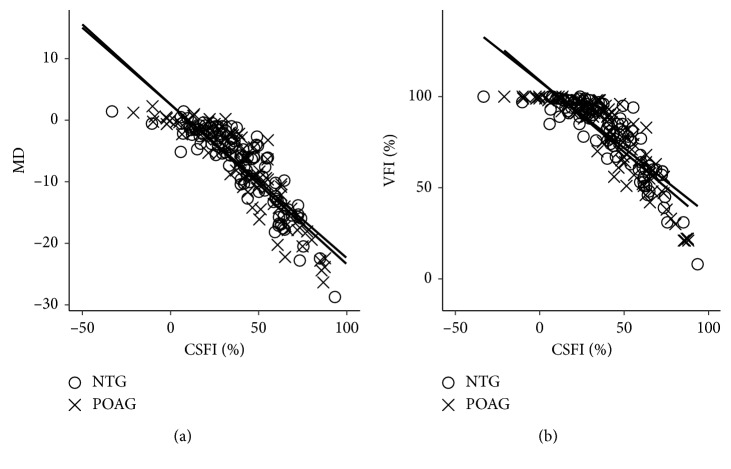
Effect on relationship of CSFI with MD and VFI by glaucoma type of disease. The effect of both CSFI and MD or VFI on the glaucoma type of disease was examined using analysis of covariance (ANCOVA). (a) Relationship between CSFI and MD in POAG and NTG: *F* = 0.27; *p*=0.60. (b) Relationship between CSFI and VFI in POAG and NTG: *F* = 0.66; *p*=0.42.

The effect of both CSFI and MD or VFI on the glaucoma type of disease was examined using ANCOVA and was unaffected by the disease type (Figure 4).

### 3.4. Correspondence between SAP rgc and OCT rgc

**Figure 5:**
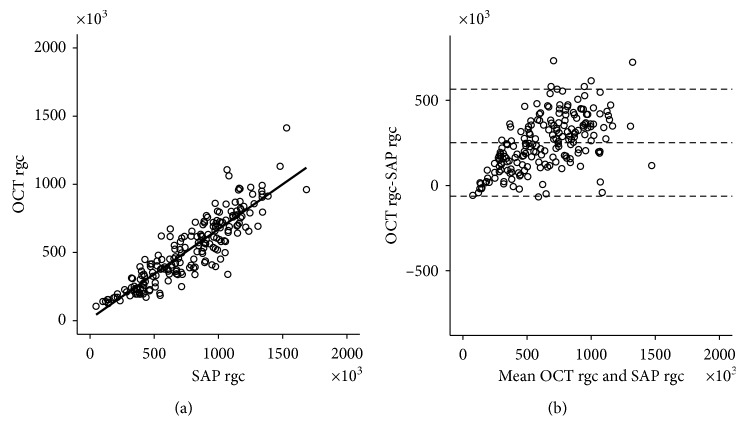
Correspondence between SAP rgc and OCT rgc. The relationship between SAP rgc and OCT rgc was evaluated using Bland–Altman analysis. (a) Correlation between SAP rgc and OCT rgc: *R*^2^ = 0.7981; *r* = 0.893; *p* < 0.001; *y* = 0.66*x* + 12.38. (b) Bland–Altman analysis of SAP rgc and OCT rgc. Proportional error was confirmed by Bland–Altman analysis (*t* = 27.622; degrees of freedom = 193). Dotted lines indicate mean and ± 1.96 standard deviation from mean (95% CI).

Bland–Altman plots of the relationship between SAP rgc and OCT rgc are shown in Figure 5. We found a significant correlation between SAP rgc and OCT rgc: *R*^2^ = 0.7981, *r* = 0.893, *p* < 0.001. However, proportional bias was confirmed by Bland–Altman analysis [19] (*t* = 27.622, degrees of freedom = 193) (Figure 5). The existence of proportional bias indicates that the two methods do not agree equally through the range of measurements. The limits of agreement will depend on the actual measurement. But, we do not know the actual measurement (actual number of RGC). The method only defines the intervals of agreements; it does not say whether those limits are acceptable or not [20].

## 4. Discussion

A discrepancy was seen in the timing of the structural changes in glaucoma detectable on OCT and the functional changes detectable with a static visual field analyzer. The nature of perimetry means that reproducibility is poor. Patients with advanced glaucoma exhibit a high degree of variability, with a coefficient of variance of 13–28% [22]. Multiple tests are thus required to detect the advance of the disease. Conversely, OCT measurements were highly reproducible, as both NFL and GCC showed coefficients of variance of approximately 3%, but as these values measure the thickness of retinal layers, the rate of decrease slowed down from the middle stage onward, meaning that these values are less able to detect progress in the advanced stage. Zhang et al. [23] used OCT and perimetry to assess progression in glaucoma suspects (GS), preperimetric glaucoma (PPG), and perimetric glaucoma in 356 patients. OCT was superior to perimetry in patients with PPG and GS. Investigation by PG stage found that OCT was superior in the early stage, but no significant differences were evident in the middle or advanced stages. Structural changes in glaucoma thus preceded functional changes [10, 24], and studies have found that, by the time visual field abnormalities are detectable on static perimetry, 20–50% of the RGC is already damaged [14, 24, 25].

As described by Medeiros et al. [5], CSFI calculates the proportion of RGC remaining in the retina by combining the results of SAP and OCT measurements, enabling the use of the advantages of SAP and OCT at all disease stages. CSFI thus provides a single index for evaluating all stages of glaucoma. A number of studies have reported the value of using CSFI at all stages of glaucoma, and this study also identified a strong correlation between MD and VFI at each disease stage. However, previous reports have not fully investigated either NTG or POAG in CSFI by the disease type or its value in individual disease stages.

This study investigated the association between CSFI and MD or VFI, as representative indices of the severity of visual field impairment in SAP, in each stage of glaucoma. Our investigation of the association between CSFI and MD or VFI at each stage showed that although CSFI correlated significantly and strongly with both MD and VFI during the early and advanced stages, this correlation was not significant in the middle stages. Differences in NTG and POAG disease types had no effect on the correlations between CSFI and MD or VFI. Because the formula for calculating CSFI itself includes MD, the correlation with MD was expected to be stronger than that with VFI. However, the correlation between CSFI and VFI was only stronger than that with MD in the advanced stage (Figure 3). This may be due to the formulae for calculating MD and VFI. MD is calculated from total deviation in all stages of disease, whereas VFI is calculated from pattern deviation at MD ≥ −20 dB and from total deviation at < −20 dB. This takes account of the cerebral cortical magnification rate and the distribution of retinal neurons and is weighted toward the clinically important central visual field [26].

We therefore divided eyes in the advanced stage according to MD about a cutoff of −20 dB (−12 dB > MD ≥ −20 dB, 37 eyes; MD < −20 dB, 12 eyes) and further investigated the correlation of CSFI with MD and VFI. For eyes with MD ≥ −20 dB, CSFI correlated moderately with MD and VFI (MD: *r* = −0.47, *p*=0.003; VFI: *r* = −0.57, *p*=0.0002), whereas the correlation was strong for eyes with MD < −20 dB (MD: *r* = −0.71, *p*=0.01; VFI: *r* = −0.95, *p* < 0.001). CSFI is thus less consistent with VFI when MD as measured by Humphrey field analysis (HFA) is between −6 dB and −20 dB. This may be due to the effect of the redundancy of RGC in OCT and variability in visual field measurements in HFA. However, further investigations of an increased number of advanced cases are required to clarify why VFI, which is weighted toward the central visual field, appears preferable for this stage. Next, SAP rgc and OCT rgc correlated strongly in broadly defined POAG (Figure 5). However, proportional error was evident in the association between these parameters. Evidence for the calculation of SAP rgc and OCT rgc is based on the monkey experiments of Harwerth et al. [27], and Medeiros et al. [5] introduced a weighting into the Harwerth model for use in the clinical management of human glaucoma. However anatomically close monkeys may be to humans, human studies are ultimately necessary. One example, although not included in this study, is the finding by Drasdo et al. [28] that taking account of RGC displacement further improves the association between SAP rgc and OCT rgc. Future improvements in OCT resolution and advances in SAP measurement techniques may further refine the model for estimating the number of RGCs.

The main limitation of this study was that measurement errors were included in both OCT and HFA measurements. Factors affecting the measurement of cpRNFL by OCT include individual differences in the papillae and their surrounding structures, age-related changes in large blood vessel caliber [29], and the effects of axial length [30, 31]. HFA measurements have problems with reproducibility due to the subjective nature of this test. Studies have found that, for the same measurement program, variation is greater at the periphery than in the center [32, 33]. A second limitation was that the study only included patients with broadly defined OAG. The formula for calculating CSFI itself calculates the number of RGCs by adapting the monkey glaucoma model of Harwerth et al. [27] to the size of the human eyeball. Glaucoma stage, corrected by age and MD, was used to optimize the model for glaucoma (see the calculation formula in Section 2), making this a model designed specifically for glaucoma patients. Further studies including healthy individuals are required.

A recent study found that visual field abnormality detected by visual field measurements made with a 2° grid was frequently assessed as normal by visual field measurements made with a 6° grid and recognized the value of HFA10-2 [34]. In addition, more in-depth studies of the detailed correspondence between function and structure that includes healthy individuals and uses perimetry with a 2° grid within 10° of the center may be useful to improve the association between structure and function [35–37]. The method described by Medeiros et al. of examining SAP and OCT results in terms of the number of RGCs [5] is clinically valuable. Advances in our understanding of the human retina should lead to further improvements.

We investigated the association of MD and VFI with CSFI as an indicator of the glaucoma stage. CSFI correlated strongly with MD and VFI in the overall study population. However, analysis by disease stage showed that this correlation was weaker in the middle stage than in either the early or advanced stage. The variation in visual field severity evaluation by CSFI in the middle stage means that caution is required when using it as an assessment tool. This should be taken into account in assessing the results, which should be considered in combination with other methods of evaluating progression.

## 5. Conclusions

This work investigated the relationship between CSFI and SAP parameters. Based on these results, caution is required when analyzing the CSFI in middle-stage glaucoma even though CSFI offers a good index of the evaluation of visual field severity at different stages of glaucoma.

## Figures and Tables

**Table 1:** Clinical characteristics.

Parameter	All (*n* = 195)	Early (*n* = 104)	Middle (*n* = 42)	Advanced (*n* = 49)	*p* value
Age (years)^*∗*^	61.4 ± 11.3	61.6 ± 11.2	62.4 ± 10.6	60.1 ± 12.2	0.60
SE (D)^*∗*^	−2.39 ± 2.28	−1.98 ± 2.19	−2.57 ± 2.50	−3.11 ± 2.09	0.01^#^
Sex (male : female)^*∗∗*^	128 : 67	63 : 41	28 : 14	37 : 12	0.18
MD (dB)^*∗*^	−7.38 ± 6.84	−2.13 ± 1.99	−8.78 ± 1.78	−17.31 ± 3.90	<0.001^#^
VFI (%)^*∗*^	78.7 ± 21.0	94.0 ± 5.5	75.4 ± 8.7	49.0 ± 15.2	<0.001^#^
CSFI (%)^*∗*^	39.4 ± 23.7	22.4 ± 16.2	47.9 ± 7.7	68.3 ± 10.9	<0.001^#^

**Table 2:** Comparison of glaucoma types.

Parameter	POAG (*n* = 99)	NTG (*n* = 96)	*p* value
Age (years)^*∗*^	61.9 ± 12.2	60.8 ± 10.4	0.37
SE (D)^*∗*^	−2.45 ± 2.35	−2.33 ± 2.20	0.68
Sex (male : female)^*∗∗*^	68 : 31	60 : 30	N.S.
MD (dB)^*∗*^	−7.53 ± 7.24	−7.21 ± 6.43	0.93
VFI (%)^*∗*^	78.0 ± 22.6	79.4 ± 19.3	0.90
CSFI (%)^*∗*^	39.3 ± 25.0	39.5 ± 22.3	0.98

## Data Availability

The data used to support the findings of this study are restricted by the Jikei University Hospital Ethics Committee in order to protect patient privacy. Data are available from Yoshimasa Tanabe and Shumpei Ogawa for researchers who meet the criteria for access to confidential data.
